# Hypoglycemic effect of *C. butyricum*-pMTL007-GLP-1 engineered probiotics on type 2 diabetes mellitus

**DOI:** 10.1080/19490976.2024.2447814

**Published:** 2025-01-02

**Authors:** Dexi Zhou, Shengjie Li, Gang Hu, Yufan Wang, Zhanghua Qi, Xuan Xu, Jing Wei, Qiong Liu, Tingtao Chen

**Affiliations:** a School of Pharmacy, Jiangxi Medical College, Nanchang University, Nanchang, China; b National Engineering Research Center for Bioengineering Drugs and the Technologies, Institute of Translational Medicine, Jiangxi Medical College, Nanchang University, Nanchang, China; c Department of Medical Microbiology, School of Medicine, Jiangxi Medical College, Nanchang University, Nanchang, China; d The MOE Basic Research and Innovation Center for the Targeted Therapeutics of Solid Tumors, Jiangxi Medical College, Nanchang University, Nanchang, China

**Keywords:** Type 2 diabetes mellitus, *Clostridium butyricum*, GLP-1, engineering probiotics, insulin secretion, gut microbiota

## Abstract

Diabetes mellitus (DM) is a complex metabolic disease characterized by hyperglycemia. Recently, the incidence of diabetes has increased exponentially, and it is estimated to become the seventh leading cause of global mortality by 2030. Glucagon-like peptide-1 (GLP-1), a hormone derived from the intestine, has been demonstrated to exert remarkable hypoglycemic effects. However, its limitation lies in its short plasma half-life, necessitating the continuous intravenous injection of GLP-1 drugs to achieve efficacy. Here, we engineered *Clostridium butyricum* to continuously express and deliver GLP-1 (denoted as Cb-GLP-1), and assessed its therapeutic efficacy in type 2 diabetes mellitus (T2DM) mice. We demonstrated that administration of Cb-GLP-1 effectively lowered blood glucose levels, regulated dyslipidemia, and ameliorated hepatic impairment in T2DM mice. Furthermore, Cb-GLP-1 treatment facilitated insulin secretion by retarding islet cell apoptosis and activating the glucagon-like peptide 1 receptor/adenylate cyclase/protein kinase A (GLP-1 R/AC/PKA) signaling pathway. Gut microbiota analysis revealed that Cb-GLP-1 restored gut homeostasis disrupted in T2DM mice, as indicated by the decreased abundance of *Lactobacillus* and *Providencia* genera in response to Cb-GLP-1 treatment. Collectively, the intestinal microbiota regulation and hypoglycemic effect of the engineered strain Cb-GLP-1 presents a promising approach for diabetes management.

## GRAPHICAL ABSTRACT

**Figure:**
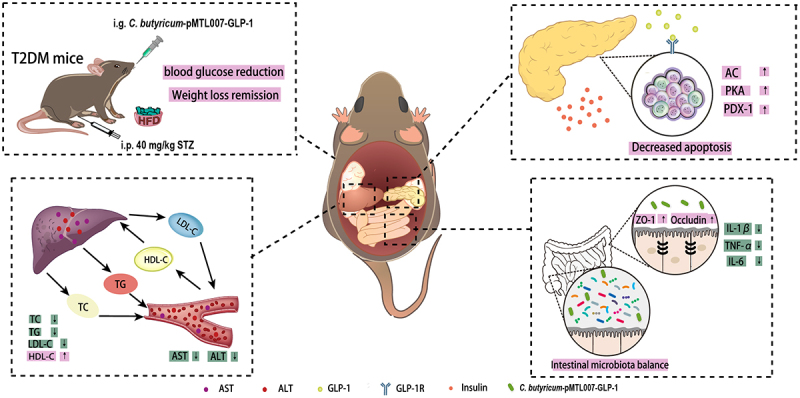

## Introduction

Type 2 diabetes mellitus (T2DM) is a global epidemic resulting from the intricate interplay between genetic background and environmental factors. It has been demonstrated that T2DM is characterized by the progressive dysfunction of pancreatic beta-cells and varying degrees of insulin resistance.^1^ According to the latest Global Diabetes Map from the International Diabetes Federation (IDF), the worldwide number of individuals aged 20–79 with diabetes reached approximately 536.6 million in 2021, and this number is expected to climb to 783.2 million by 2045, with T2DM accounting for 90% to 95% of these cases.^2,3^ This chronic disease can lead to several complications, including retinopathy, kidney diseases, ischemic heart disease, and stroke, ultimately culminating in multi-organ failure and even mortality.^4,5^ While specific medications such as biguanides, sulfonylureas, thiazolidinediones, and alpha-glucosidase inhibitors play an essential role in managing T2DM, they are often accompanied by gastrointestinal complications like nausea, vomiting, and diarrhea.^6^ Therefore, it is imperative to develop innovative antidiabetic drugs that not only guarantee therapeutic efficacy but also minimize adverse effects.

Glucagon-like peptide-1 (GLP-1), a peptide hormone secreted by intestinal L-cells, exhibits hypoglycemic effects dependent on glucose concentration. It has been reported that GLP-1 could retard the progression of T2DM by promoting the proliferation and differentiation of pancreatic beta-cells while inhibiting their apoptosis.^7^ However, natural GLP-1 is rapidly inactivated by dipeptidyl peptidase-IV (DPP-IV) proteolysis *in vivo* and ultimately cleared by the kidney, resulting in a brief half-life of only 1–2 minutes, significantly limiting its clinical application.^8^ To address this challenge, medications such as DPP-IV inhibitors and GLP-1 analogs have been widely explored and adopted.^9^ However, issues like high drug prices and the necessity for long-term injections persistently affect the majority of individuals diagnosed with T2DM. Consequently, it is of great practical significance to explore GLP-1 delivery routes with low economic cost and strong operability.

Mounting studies have unveiled a connection between the dysbiosis of gut microbiota and the progression of diabetes.^10^ A clinical study showed that the proportions of Firmicutes and Clostridia (belonging to phylum Firmicutes) were significantly reduced, while that of Bacteroidetes was increased, in the gut of T2DM patients.^11^ A metabolome Genome-Wide Association Study (mGWAS) highlighted that the gut microbiota of T2DM patients was predominantly enriched with opportunistic pathogens like *Escherichia coli*, while the abundance of butyrate-yielding bacteria was reduced.^12^ These findings illustrate the potential of targeting the intestinal microbiota as a therapeutic approach for T2DM.

*Clostridium butyricum*, a strictly anaerobic gram-positive bacillus, is well known for its ability to produce butyric acid, thereby possessing valuable probiotic properties for intestinal homeostasis and health promoting. At the same time, it can form spores in the later stages of growth to tolerate harsh environments, such as strong acids, high temperatures, and high salts for their survival. It has been reported that supplementing *C. butyricum* in a diabetic mouse model could reduce weight gain and fat accumulation in mice, as well as improve glucose tolerance and insulin sensitivity.^13^ Additionally, oral administration of *C. butyricum* CB0313.1 has been shown to attenuate insulitis and inhibit the occurrence of diabetes in non-obese diabetic (NOD) mice.^14^ In other words, increasing the abundance of butyrate-yielding bacteria or improving the ability of gut bacteria to synthesize butyrate may be an important part of the diabetes management. Furthermore, engineering *C. butyricum* for exogenous drugs (e.g., GLP-1) delivery might be more interesting in treating and preventing diabetes, taking the advantage of the synergies between drugs and *C. butyricum* or its-produced butyrate *in vivo*.

Prior to this study, we successfully engineered a *C. butyricum* strain to effectively express and deliver GLP-1 using the recombinant shuttle plasmid pMTL007-GLP-1. We demonstrated that the engineering strain showed remarkedly improvements in spontaneous hypertension rat model and Parkinson’s disease mice model, respectively.^15,16^ Here, we investigated the hypoglycemic effects of the engineered strain on T2DM mice and elucidated their underlying mechanisms through a comprehensive approach that included blood glucose monitoring, biochemical analysis, protein immunoblotting, histopathology examination, and high-throughput 16S rRNA sequencing of gut microbiota. We anticipate that this study will offer innovative strategies and measures for the clinical management of diabetes, providing valuable data to propel the future development of engineered bacterial drugs.

## Materials and methods

### Experimental strains and plasmids

*C. butyricum* (CGMCC, No. 25504) was isolated from the feces of a healthy volunteer in Jiangxi Province, China (written consent was obtained from the volunteer). *E. coli* CA434 and plasmid pMTL007 were obtained from Prof. Hongjun Dong of Tianjin Institute of Industrial Biotechnology, Chinese Academy of Sciences. The GLP-1 gene was integrated into the 5′ *Hin*dIII to 3′ *BsrGI* end of the pMTL007 plasmid (GenBank: EF525477.1) to obtain the recombinant plasmid pMTL007-GLP-1. This study was entrusted to GENEWIZ Biotechnology Co., Ltd. (Suzhou, China).

### *The construction and characterization of* C. butyricum-*pMTL007-GLP-1 engineered strain*

The plasmid pMTL007-GLP-1 was transferred into the donor strain *E. coli* CA434 by thermal excitation, and then into *C. butyricum* after splicing with *E. coli* CA434. To obtain positive clones, transfected colonies were restrained on TSA plates containing 15 μg/mL thiamphenicol and 250 μg/mL D-cycloserine. Single colonies were picked from the delineated plates, inoculated in TSB medium supplemented with 15 μg/mL thiamphenicol, and incubated anaerobically at 37°С for 36–48 h. GLP-1 expression in the supernatant was detected using a human GLP-1 enzyme-linked immunosorbent assay (ELISA) kit (ELabscience, E-EL-H6025). Seven SCFAs in bacterial culture supernatants, namely acetic, propionic, isobutyric, butyric, isovaleric, valeric, and hexanoic acids, were determined using targeted metabolomics. Bacterial growth was monitored by a turbidimetric assay, whereas the bacterial acid and bile salt resistance was assessed after co-incubation with graduated pH values (pH = 3, 4, 5, 6, 7) and bile salt concentrations (0, 0.1%, 0.3%, 0.5%) for 3 hours.

### Experimental design of T2DM models and animal handling

This study and animal experimental protocols were reviewed and approved by the Laboratory Animal Ethics Committee of Nanchang Royo Biotechnology Co., Ltd., Nanchang, China (approval number: RYE2021071801).

C57BL/6 male mice (8 weeks old, weighing 20–22 g) were procured from Hunan Slaughter Kingda Experimental Animal Co. (Changsha, China) and subsequently raised in a specific pathogen-free (SPF) environment (temperature 22 ± 2°C, humidity 50 ± 15%, light/dark cycle 12/12) with ad libitum access to water. Prior to inducing Type 2 Diabetes Mellitus (T2DM), all mice underwent a one-week acclimatization period with normal chow. The 12 mice in the control group (C group) were given a standard diet, whereas the remaining mice underwent continuous intraperitoneal injection of 40 mg/kg streptozotocin (STZ) for 5 consecutive days following 12 weeks of being fed a high-fat diet (HFD) with 60% Calories from Fat and 20% from proteins (product code: XTHF60, Jiangsu Xietong Pharmaceutical Bio-engineering Co., Ltd., China). Following the modeling phase, blood glucose levels in mice were monitored every 3 days. Mice with random blood glucose levels ≥16.7 mmol/L and remaining stable were selected and randomly assigned to four groups (12 mice each): the model group (M group), wild-type *C. butyricum* group (Cb group), exenatide group (E group), and engineered *C. butyricum*-pMTL007-GLP-1 group (Cb-GLP-1 group). Mice in C and M groups were orally administered 100 μL sterile saline daily, and mice in Cb and Cb-GLP-1 groups received daily oral administration of 100 μL 10^8^ CFU/mL of the corresponding bacterial solution as well. Mice in E group was intraperitoneally injected with 24 nmol/kg exenatide, a GLP-1 analog that has been approved for the treatment of T2DM, considering as a positive control group. Except for the E group that was injected intraperitoneally with exenatide daily, the other groups were orally administered with the corresponding agent by gavage. Throughout the 10-week treatment period, changes in blood glucose and body weight were monitored every week.

### Oral glucose tolerance test (OGTT) and insulin tolerance test (ITT)

OGTT was performed two weeks before the end of treatment. The mice in each group were first fasted for 16 h and then administered 2 g/kg glucose oral liquids according to the weight of the mice. Tail vein sampling was used to test the fluctuations of mouse blood glucose at time points of 0, 30, 60, 90, and 120 min using a glucometer, thereby calculating the area under the curve (AUC) of glucose tolerance in mice. The ITT experiment was performed one week before the end of treatment. After starvation for 6 h, mice in each group received an intraperitoneal injection of 1 U/kg human insulin. Blood glucose monitoring was performed as previously described.

### Blood biochemical analysis

Mice were anesthetized with 2% isoflurane, and blood was collected by removing the eyeballs. To obtain serum, fresh blood samples were incubated at room temperature for 2 h and then centrifuged at 3500 × g for 15 min. Serum levels of total cholesterol (TC), triglyceride (TG), low-density lipoprotein (LDL-C), high-density lipoprotein (HDL-C), aspartate aminotransferase (AST) and alanine aminotransferase (ALT) were determined according to the kit instructions from Nanjing Jiancheng Biotechnology Co. (Nanjing, China) (Product No. A111-1-1, A110-1-1, A113-1-1, A112-1-1, C010-2-1, C009-2-1).

### Histopathological tests

Pancreatic tissue samples were fixed, embedded, and sliced into 5 μm thick sections for subsequent H&E and immunofluorescence staining. For immunofluorescence staining, paraffin sections were deparaffinized with water, antigenically repaired, and incubated with insulin (#4590S; Cell Signaling Technology, USA) primary antibody overnight (4°C) and secondary antibody for 1 h (room temperature). The images were captured using a fluorescence microscope (Mingmei Microscope MF23-M, Guangzhou, China). For liver oil red O staining, the sections were placed in oil-red stain in the dark for 15 min and sequentially rinsed with 60% isopropyl alcohol solution and double-distilled water. After hematoxylin staining for 2 min, sections were washed, blocked with glycerol, and visualized under an optical microscope (NIS-Elements 3.2, Nikon Eclipse 80i, Nikon, Japan). Immunofluorescence staining and oil red O staining results were quantitatively analyzed using Image J software (v1.8.0, National Institutes of Health).

### Western blot

Each fresh tissue (~100 mg) was lysed in 1 mL of high-efficiency RIPA solution containing phenylmethylsulfonyl fluoride (PMSF) protease inhibitor and homogenized using an ultrasonic breaker on ice. The resulting protein mixture was then obtained by centrifugation at 12,000 rpm for 30 min at 4°C. The obtained supernatant was used to determine the protein concentrations by bicinchoninic acid (BCA) method, followed by sodium dodecyl sulfate polyacrylamide gel electrophoresis, membrane translocation, and milk containment solution closure for 1 h, TBST washing, and antibody incubation procedure, as mentioned above. The images were captured in the gel imaging analysis system and the grayscale values of the strips were determined. See Table S1 for details of the antibodies.

### Real-time fluorescence quantitative PCR

After extracting colonic tissue total RNA with Trizol reagent, the RNA was reverse-transcribed to cDNA using the PrimeScript® RT kit, preparing for further PCR amplification using the SYBR® Premix Ex Taq™ II kit. The qPCR parameters were as follows: denaturation (95°C, 30 s), annealing (60°C, 30 s), extension (72°C, 30 s), and 40 cycles of circulating. The relative expression levels of the described RNAs were normalized to that of GAPDH using the 2^−ΔΔCt^ method. See Table S2 for primer details.

### 16S rRNA high-throughput sequencing

The DNA of the collected mouse fecal samples was extracted with reference to the instruction manual of the Tiangen Bacterial Genomic DNA Extraction Kit, and the V4 region of 16S rDNA was amplified using general barcoded primers 515F (5’-AYTGGGYDTAAAGNG-3’) and 806 R (5’-TACNVGGGTATCTAATCC-3’), and then sent to Shanghai Paisano Biotechnology Co. (Shanghai, China) for high-throughput sequencing. The raw sequences were optimized using Quantitative Insights into Microbial Ecology (QIIME2) (v 2019.4). The database of Greengenes (Release 13.8, http://greengenes.secondgenome.com/), a commonly selected database for high-throughput sequencing analysis, was used to denote the sequences. the analyses included microbial species and relative abundance, α-diversity index, β-diversity analysis, and inter-group differences. High-throughput sequencing data have been submitted to NCBI (GenBank accession number PRJNA1018089).

### Statistical analysis

All data were analyzed using GraphPad Prism statistical software (the 8^th^ version of prism, https://www.graphpad.com/). Student’s t-test was employed to discern differences between two independent samples, while one-way or two-way analysis of variance (ANOVA) combined with Tukey’s multiple comparisons were utilized to identify statistical differences among three groups or more. Data were presented as the mean ± standard deviation (SD). *p < 0.05 or **p < 0.01 denoted a statistically significant difference for the comparison between two samples.

## Results

### *Characterization of the engineered Cb-GLP-1 strain* in vitro

**Figure 1.**
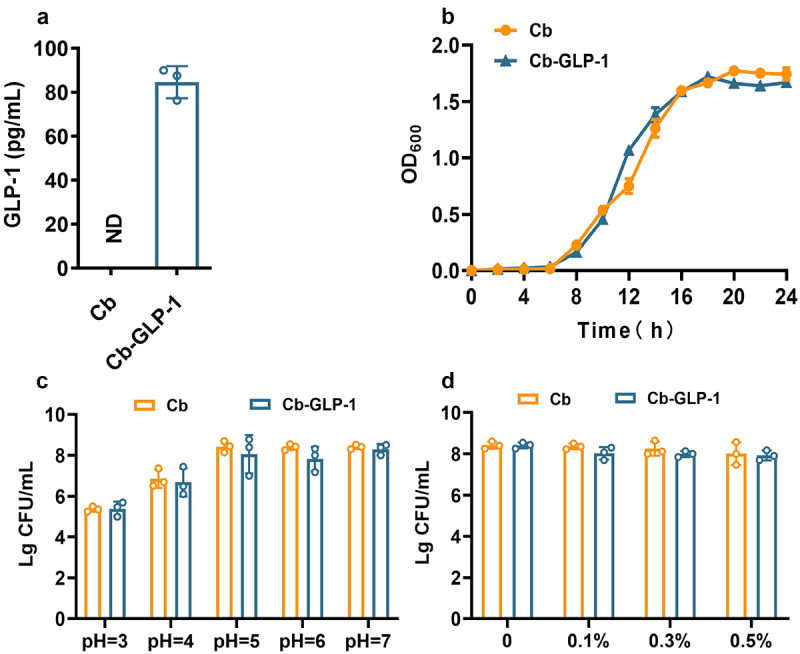
Assessment of GLP-1 expression capability of the engineered strain *C. butyricum*-pMTL007-GLP-1 and its probiotic characteristics *in vitro*. (a) ELISA analysis of the expression and secretion of GLP-1 on the supernatant of Cb and Cb-GLP-1 strains; (b) growth curves of Cb and Cb-GLP-1; (c) the acid resistance of Cb and Cb-GLP-1; (d) bile salt tolerance of Cb and Cb-GLP-1. Cb, *C. butyricum* wild-type strain; Cb-GLP-1, *C. butyricum*-pMTL007-GLP-1 engineered strain. Three parallel experiments were performed for each assay. Data were presented as means ± SD. Compared with Cb (t-test), no statistical difference annotation means no significant difference (*p* > 0.05).

The results showed that GLP-1 expression in the Cb-GLP-1 strain reached a significantly higher level of 84.58 pg/mL (Figure 1(a)). Targeted metabolomic analysis showed no disparity in their SCFAs production (Figure S1). The growth curves suggested that the expression of GLP-1 in the Cb-GLP-1 strain had no impact on its growth, as both strains entered the logarithmic growth phase after incubation for 8 hours and achieved stationary phase after 16 hours (Figure 1(b)). Additionally, both strains exhibited excellent acid and bile salt tolerance at different pH values and bile salt concentrations (Figure 1(c,d)). These results were consistent with our published data, suggesting a robust genetic stability of this engineered strain.^15,16^

### Cb-GLP-1 improved the blood glucose, body weight, glucose tolerance and insulin tolerance in T2DM mice

**Figure 2.**
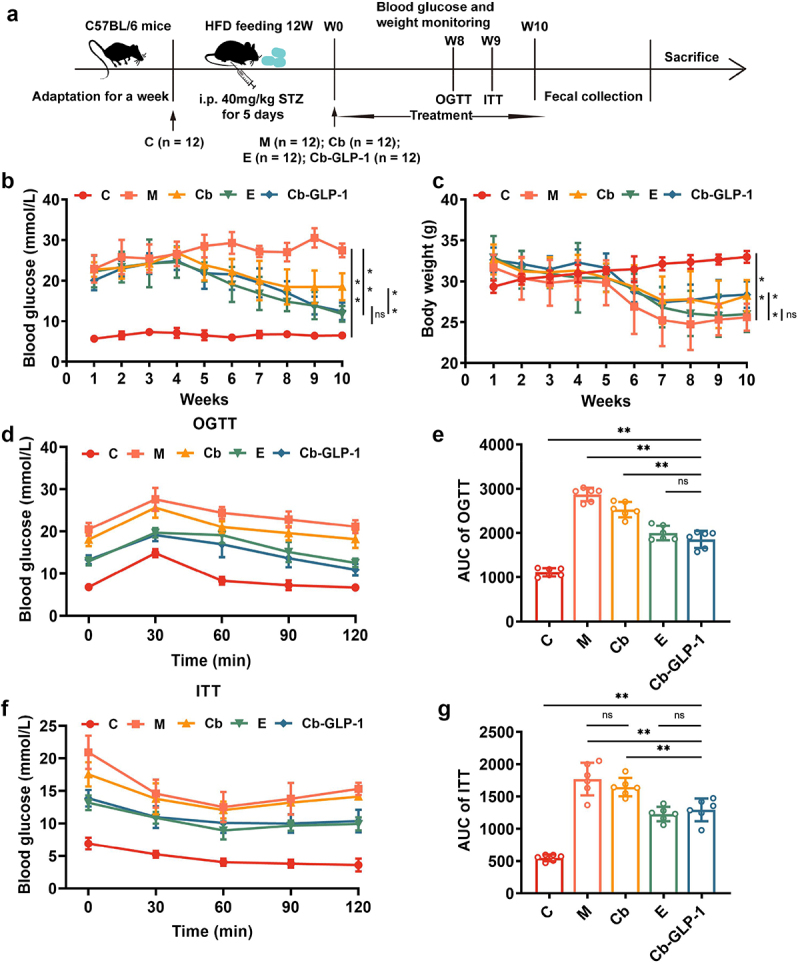
Cb-GLP-1 improved hyperglycemic symptom and prevented weight loss in T2DM mice. (a) The animal experiment flow chart of this study; (b) detection of blood glucose levels in mice during treatment; (c) monitoring the mice body weight changes during treatment; (d) mice oral glucose tolerance test (OGTT) and (e) its area under the curve (AUC) analysis after treatment; (f) mice insulin tolerance test (ITT) and (g) its area under the curve (AUC) analysis after treatment. C group, the normal control mice treated with sterile saline; M group, the T2DM model mice treated with sterile saline; Cb group, the T2DM mice treated with *C. butyricum*; E group, the T2DM mice treated with exenatide; Cb-GLP-1 group, the T2DM mice treated with the engineered probiotic of *C. butyricum*-pMTL007-GLP-1. Data were presented as means ± SD, calculated from a random selection of 6 mice/group each time. Two-way ANOVA analysis was conducted for (b) and (c), while one-way ANOVA analysis for (e) and (g), both followed by Tukey’s multiple comparison. *p < 0.05, **p < 0.01, ns or no mark indicate no significant difference (*p* > 0.05).

We evaluated the ameliorative effect of Cb-GLP-1 on T2DM mouse modeling. As shown in Figure 2(b), after treated with *C. butyricum* (Cb group), exenatide (E group), and *C. butyricum*-pMTL007-GLP-1 engineered bacteria (Cb-GLP-1 group), mice exhibited a gradual reduction in blood glucose levels compared to the model ones (M group); the most pronounced therapeutic effect was observed in the Cb-GLP-1 group (M *vs*. Cb-GLP-1, 28.5 *vs*. 21.85, *p* < 0.01). The Cb-GLP-1 group continued to exhibit a declining trend in blood glucose levels from week 5 until the end of treatment, with a glucose-lowering effect similar to the positive drug. Meanwhile, mice in the M group exhibited a noticeable decrease in body weight compared with the normal control ones (C group), yet the treatment of Cb and Cb-GLP-1 significantly attenuated the extent of body weight loss, although the body weight of mice in all other groups declined at varying degrees (Figure 2(c)). Ultimately, the results of the OGTT and ITT suggested that Cb-GLP-1 treatment markedly enhanced glucose tolerance and improved insulin sensitivity compared to the M group (Figure 2(d–g)). Therefore, treatment with the engineered Cb-GLP-1 strain significantly alleviated hyperglycemia and prevented weight reduction in T2DM mice, providing key data for further elucidation of the underlying molecular mechanisms.

### Cb-GLP-1 regulated dyslipidemia, ameliorated hepatic impairment and reduced lipid deposition in T2DM mice

**Figure 3.**
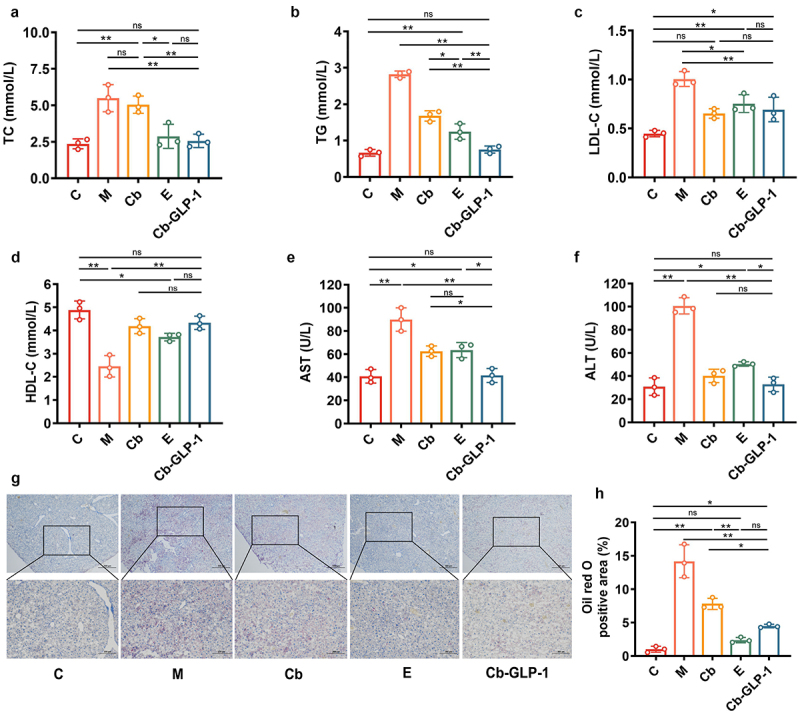
Cb-GLP-1 mitigated dyslipidemia, alleviated liver injury, and diminished lipid deposition in T2DM mice. (a-f) the quantitative analysis of total cholesterol (TC), triglycerides (TG), low-density lipoprotein (LDL-C), high-density lipoprotein (HDL-C), aspartate transaminase (AST), and alanine transaminase (ALT) in the liver of mice among C, M, Cb, E, and Cb-GLP-1 groups; (g) liver oil red O staining (40 × and 100 ×) and (h) its relative area of lipid droplets. C, normal control group; M, the T2DM model group; Cb, the *C. butyricum* group; E, the exenatide group; Cb-GLP-1, the *C. butyricum*-pMTL007-GLP-1 engineered stain group. Data are presented as means ± SD, calculated from a random selection of 3 mice/group (*n* = 3). One-way ANOVA combined with Tukey’s multiple comparisons test was conducted for the statistical analysis, *p < 0.05 and **p < 0.01, ns or no mark indicate no significant difference (*p* > 0.05).

As shown in Figure 3(a), the Cb-GLP-1 group exhibited a significant reduction in serum TC concentration compared to the M group (M *vs*. Cb-GLP-1, 5.497 *vs*. 2.566, *p* < 0.01). Mice in the M group showed elevated serum TG levels compared to those in group C, and TG levels in the three treatment groups were reduced compared to those in group M, with reductions of 40.56%, 55.83%, and 73.2% in the Cb, E, and Cb-GLP-1 groups, respectively (Figure 3(b)).

As depicted in Figure 3(c), the differences in LDL-C levels among the groups were similar to the results of TC and TG, which remained the highest in group M. The LDL-C levels in the Cb (0.6544 *vs*. 1.006, *p* < 0.01), E (0.7555 *vs*. 1.006, *p* < 0.05), and Cb-GLP-1 (0.694 *vs*. 1.006, *p* < 0.01) groups were significantly lower than those in the M group. However, no remarkable differences were found between the Cb, E, and Cb-GLP-1 groups. HDL-C was obviously lower in mice serum of group M than group C. And its concentration increased to some extent after Cb-GLP-1 administration, with a statistically significant difference observed (*p* < 0.01) (Figure 3(d)).

Furthermore, we observed that the AST and ALT levels in the Cb-GLP-1 group decreased to near-normal levels compared to those in group M (Figure 3(e,f)). Based on the results of Oil Red O staining in liver sections, T2DM mice in group M exhibited noticeable lipid accumulation compared to the C group (*p* < 0.01), characterized by prominent red oil droplets in their livers (Figure 3(g,h)). Conversely, treatment with *C. butyricum* (Cb group), exenatide (E group), and *C. butyricum*-pMTL007-GLP-1 engineered bacteria (Cb-GLP-1 group) significantly reduced the presence of oil droplets in the livers, demonstrating their hepatoprotective effects in T2DM mice (Figure 3(g,h)).

### Cb-GLP-1 prevented the occurrence of pancreatic islet cell apoptosis in T2DM mice

**Figure 4.**
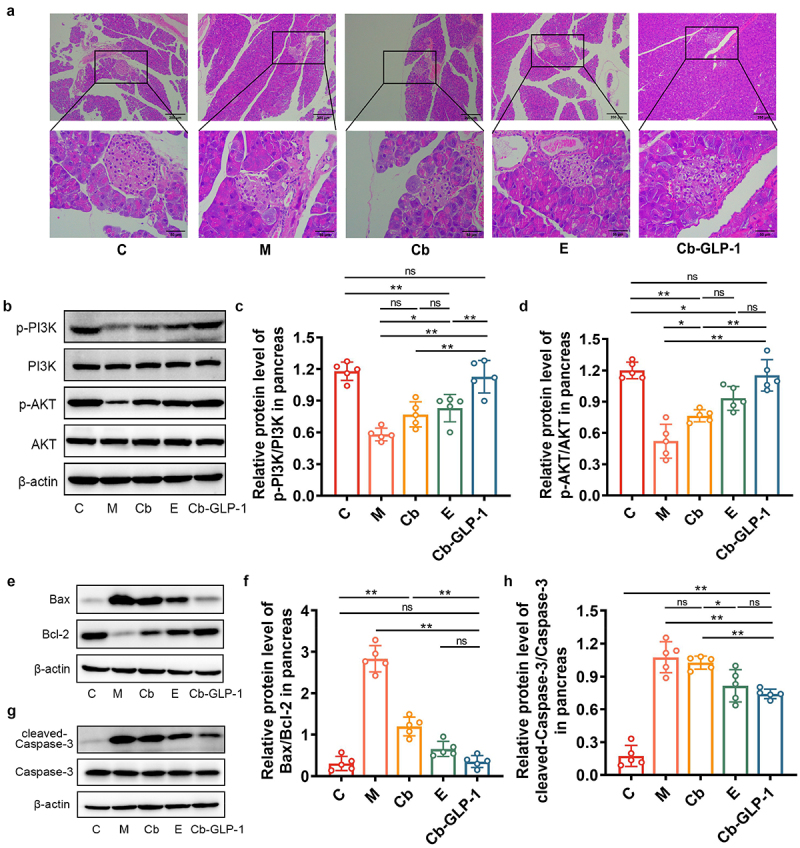
Cb-GLP-1 prevented pancreatic cell apoptosis in T2DM mice by inhibiting the expression of proteins associated with apoptotic pathway. (a) H&E staining showing the pathological changes in pancreatic tissues (100 × and 400 ×); (b-d) effects of Cb-GLP-1 on the expression of p-PI3K/PI3K and p-akt/akt in pancreatic tissues in T2DM mice; (e-f) effects of Cb-GLP-1 on the expression of Bax/Bcl-2 and (g-h) cleaved-caspase-3/Caspase-3 in pancreatic tissues in T2DM mice. C, normal control group; M, the T2DM model group; Cb, the *C. butyricum* group; E, the exenatide group; Cb-GLP-1, the *C. butyricum*-pMTL007-GLP-1 engineered stain group. Data were presented as means ± SD (*n* = 5 per each group). One-way ANOVA compared with Tukey’s multiple comparisons test was conducted for the statistical analysis. *p < 0.05, **p < 0.01.

Previous studies have illustrated that the apoptosis of pancreatic beta cells is an impact factor in promoting the progression of diabetes mellitus.^17,18^ As shown in Figure 4(a), mouse pancreatic tissues in group M presented obvious lesions, as indicated by the unclear boundary between islet cells and exocrine glands, irregular morphology, severe vacuolization of islets, and significant reduction in islet cell number. Although there was still some vacuolization in the Cb-GLP-1 group, the islets were structurally intact, islet cells were relatively neatly arranged and increased in number, and a distinct border began to form with the exocrine gland.

Western blot results indicated a significant downregulation in the levels of phosphorylated phosphoinositide 3 kinase (p-PI3K)/PI3K and phosphorylated protein kinase B p-AKT/AKT in the pancreatic tissues of T2DM mice in the M group comparing to the C group (*p* < 0.01) (Figure 4(b)). This resulted in a remarkable increased Bcl-2-associated X protein/B cell lymphoma-2 (Bax/Bcl-2) ratio and enhanced cleaved-Caspase-3 expression (*p* < 0.01), ultimately exacerbating islet cell apoptosis (Figure 4(e,g)). However, the administration of *C. butyricum* (Cb), exenatide (E), and *C. butyricum-*pMTL007-GLP-1 (Cb-GLP-1) markedly reversed this tendency, with the engineered strain Cb-GLP-1 showing the best effect (Figure 4(b-h)), aligning with the H&E staining results (Figure 4(a)). In summary, the engineered Cb-GLP-1 strain exerted protective effects on the pancreas of T2DM mice to some extent, as evidenced by the inhibition of apoptosis pathway protein expression in this tissue, thereby effectively preventing apoptosis.

### Cb-GLP-1 promoted insulin secretion in pancreatic tissues of T2DM mice

**Figure 5.**
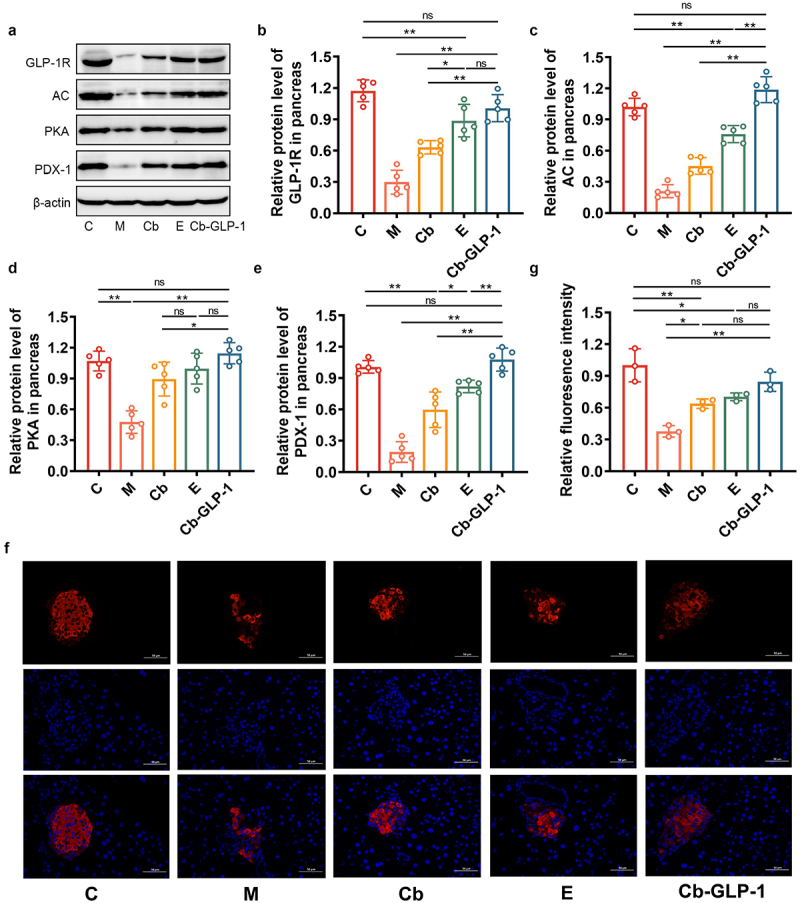
Cb-GLP-1 upregulated the expression of proteins involved in insulin secretion pathway and enhanced the insulin secretion in the pancreas of T2DM mic. (a-e) the expression of pancreatic GLP-1 R, AC, PKA, and PDX-1 proteins after treating with Cb, E, and Cb-GLP-1; (f) insulin immunofluorescence showing the expression of insulin in the pancreas (400 ×); (g) relative quantification of insulin fluorescence intensity. Data are presented as means ± SD, calculated from a random selection of 5 mice/group for western blotting analysis and 3 mice/group for immunofluorescence analysis. One-way ANOVA combined with Tukey’s multiple comparisons test was conducted for the statistical analysis, *p < 0.05 and **p < 0.01, ns or no mark indicate no significant difference (*p* > 0.05).

Studies have proposed that GLP-1 binding to the glucagon-like peptide 1 receptor (GLP-1 R) prompts insulin secretion through the activation of the insulin signaling pathway.^19,20^ In Figure 5(a-e), an obviously suppression of GLP-1 R expression in pancreatic tissues of group M was shown, while its expression was enhanced after Cb, E, and Cb-GLP-1 treatments, with the normal level gradually reached in the Cb-GLP-1 group. Similar results were observed when monitoring several proteins, including adenylate cyclase (AC), protein kinase A (PKA), and pancreatic duodenal homeobox-1 (PDX-1). Furthermore, insulin immunofluorescence experiments showed that T2DM mice exhibited significantly lower insulin expression in the pancreas compared to normal control mice (M group *vs*. C group, *p* < 0.01) (Figure 5(f,g)). Notably, treatment with *C. butyricum* (Cb group), exenatide (E group), and *C. butyricum*-pMTL007-GLP-1 engineered bacteria (Cb-GLP-1 group) substantially augmented insulin expression in the pancreas, with Cb-GLP-1 demonstrating the most notable therapeutic efficacy (Figure 5(f,g)). These results highlighted that the administration of engineered Cb-GLP-1 strain increased pancreatic insulin secretion in T2DM mice, mainly manifested as the upregulation of proteins related to the GLP-1 R/AC/PKA-modulated insulin secretion pathway.

### Cb-GLP-1 inhibited colonic inflammation and modulated intestinal microbiota balance in T2DM mice

**Figure 6.**
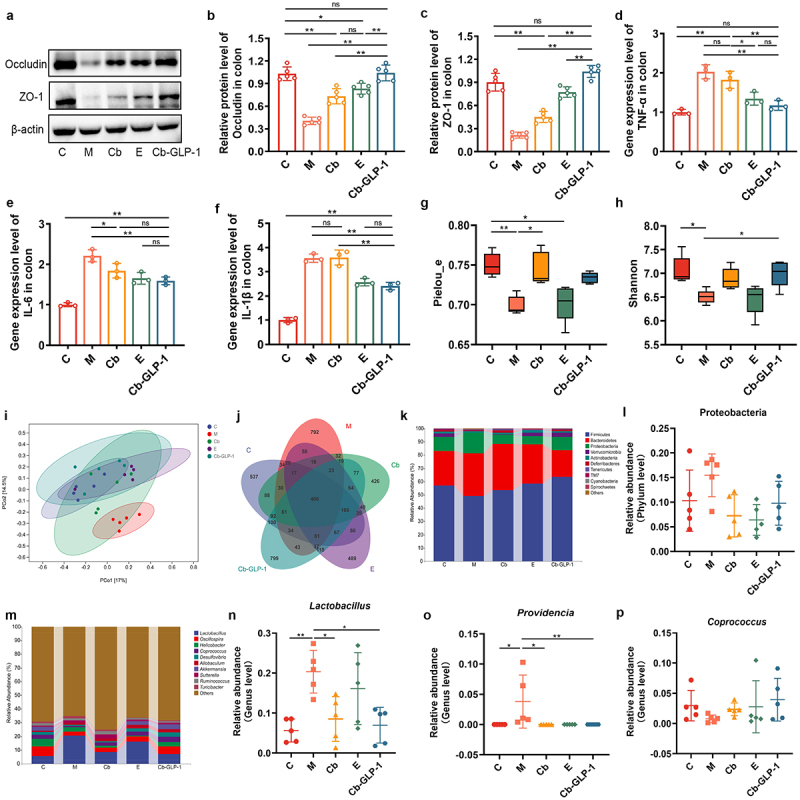
Cb-GLP-1 mitigated colonic inflammatory responses and modulated dysregulated gut microbiota. (a-c) Cb-GLP-1 enhanced the expression of Occludin and ZO-1 proteins in the colon of T2DM mice; (d-f) Cb-GLP-1 inhibited the transcription of pro-inflammatory cytokines of TNF-α, IL-6, and IL-1β in the colon of T2DM mice; (g-h) effects of cb-GLP-1 on the Pielou_e index and Shannon index of gut microbiota; (i) PCoA analysis of gut microbiota; (j) Venn diagram showing the shared and unique OTUs of gut microbiota; (k) microbial species composition analysis at the phylum level; (l) the relative abundance of proteobacteria phyla; (m) microbial species composition analysis at the genus level; the relative abundance of *Lactobacillus* (n), *Providencia* (o), and *Coprococcus* genera (p). C, normal control group; M, the T2DM model group; Cb, the *C. butyricum* group; E, the exenatide group; Cb-GLP-1, the *C. butyricum*-pMTL007-GLP-1 engineered stain group. Data are presented as means ± SD, calculated from a random selection of 5 mice/group for (a-c), 3 mice/group for (d-f), and 5 mice/group for (g-p). One-way ANOVA combined with Tukey’s multiple comparisons test was conducted for the statistical analysis of (b-f), while in the analysis of the microbiota sequencing data (g, h, I, n-p), one-way ANOVA was utilized for normally distributed non-paired data, whereas non-parametric Kruskal-Wallis tests were applied for non-normally distributed data, and multiple comparisons were conducted by Tukey test, *p < 0.05 and **p < 0.01.

The analysis of Tight junction (TJ) proteins in colon tissues by WB revealed significantly lower expression of Occludin and ZO-1 expression in the M group compared to the C group (Figure 6(a)). Notably, the treatment with Cb-GLP-1 upregulated their expression, enhancing intestinal barrier function (Figure 6(a-c)). Additionally, mice in the group M exhibited a higher inflammatory level than that of group C, as indicated by the significantly high transcription of the inflammatory factors of tumor necrosis factor-α (TNF-α), interleukin-6 (IL-6), and interleukin-1β (IL-1β) in colon tissues (Figure 6(d-f)). Interestingly, the administration of Cb-GLP-1 could effectively inhibit the T2DM-induced inflammatory level in mice (Figure 6(d-f)).

16S rRNA gene sequencing showed that group M displayed a reduced gut microbiota α-diversity compared to group C, reflected in the decline of Pielou_e index and Shannon index (Figure 6(g,h)). Principal Coordinates Analysis (PCoA) highlighted distinct community compositions between M and C groups, as indicated by the greatest distance between the sample clusters of the two groups (Figure 6(i)). However, treatment with Cb and the engineered strain Cb-GLP-1 restored fecal microbiota diversity and composition, with the Cb-GLP-1 group closely resembling group C (Figure 6(g-i)). This observation was further supported by the Venn diagram, illustrating the highest number of shared OTUs (Figure 6(j)).

Further analysis of microbiota composition at the phylum levels demonstrated a notable increase in Proteobacteria abundance in group M, while the Cb, E, and Cb-GLP-1 groups showed a slight decreasing trend (Figure 6(k,l)). At the genus level, group M exhibited significantly higher *Lactobacillus* abundance compared to group C (*p* < 0.01), with Cb and Cb-GLP-1 treatment reducing its abundance (*p* < 0.05) (Figure 6(m,n)). Moreover, an increase in *Providencia* abundance in group M was observed, distinct from C, Cb, and Cb-GLP-1 groups (*p* < 0.05) (Figure 6(o)). Additionally, *Coprococcus* abundance was slightly higher in the Cb-GLP-1 group than in the M group with no statistically significant (Figure 6(p)). Spearman correlation analysis suggested a positive correlation between changes in the abundance of *Lactobacillus* and *Providencia* with proinflammatory factors, as well as a negative correlation between *Coprococcus* and proinflammatory factors (Figure S2). These results suggested that Cb-GLP-1 treating could mitigate colonic inflammation and modulate the intestinal microbiota balance.

## Discussion

T2DM has evolved into a global challenge, imposing a substantial economic burden on society and public health.^21,22^ Although numerous synthetic drugs are available for T2DM management, their prolonged use may lead to diverse side effects such as lactic acidosis, hepatotoxicity, and gastrointestinal dysfunction.^23^ The incretin hormone GLP-1 stands out as a preferred alternative for diabetes treatment due to its exceptional incretin activity and glucose-regulatory effects. However, its short half-life poses a limitation to its practical efficacy.^24^ Therefore, the exploration of strategies to enhance the production or secretion of endogenous GLP-1 is considered a viable avenue for diabetes treatment.

Recently, probiotic therapy has gained prominence as a compelling strategy for disease treatment, propelled by a deeper understanding of the intestinal microbiota and advancements in genetic engineering. Studies have highlighted the potential use of bacteria either directly in disease treatment or as carriers to enhance drug delivery at specific sites, thereby improving therapeutic efficacy.^25^ *C. butyricum* is a well-established gut probiotic that has been safely used to prevent and treat a wide range of conditions for decades as functional foods and medicines, including gut infections, intestinal injury, irritable bowel syndrome, inflammatory bowel disease, neurodegenerative diseases, metabolic disorders, and colorectal cancer.^26^ It also has a particularly strong capacity to produce butyric acid which has been reported to have lots of health promoting functions and appositive therapeutic effects on the treatment of T2DM.^27^ Importantly, it can form spores in the later stages of growth to tolerate harsh environments, such as strong acids, high temperatures, and high salts for their survival, which facilitates the storage and shelf life of its-derived products when compared to other common probiotics. Therefore, we constructed the engineering strain Cb-GLP-1 and investigated its ameliorative effects and specific mechanisms in treating T2DM mice in this study.

We assessed the *in vitro* performance of the engineered bacteria. GLP-1 expression was detected in the supernatant of the engineered Cb-GLP-1 bacteria, with no discernible impact on the metabolism of Cb, as there were no changes in the growth capability and the ability to produce SCFAs of the engineered strain (Figure 1 and S1). Recognizing the pivotal importance of bacterial tolerance to the acidic environment in the stomach and challenging conditions of the gastrointestinal tract, particularly for survival and proliferation post-administration, our results affirmed the promising growth performance of the engineered strain and its excellent resistance to both acid and bile salts (Figure 1 and Figure S1).

Subsequently, we utilized the T2DM mice model to assess the potential impact of the engineered Cb-GLP-1 strain on mouse phenotype and histopathology. Mice were subjected to an HFD to induce a certain degree of insulin resistance, followed by low-dose STZ injections to disrupt partial function of pancreatic beta-cells to inhibit insulin secretion *in vivo*, mimicking the pathological and physiological alterations akin to human T2DM.^28^ We observed that mice in group M exhibited elevated blood glucose levels, weight loss, and impaired glucose tolerance and insulin sensitivity, which were consistent with the physiological indicators of T2DM. In contrast, these symptoms were significantly improved in the Cb-GLP-1 group, indicating an efficient therapeutic outcome of Cb-GLP-1 in treating T2DM mice (Figure 2).

One of the complications associated with diabetes is disturbed lipid metabolism. Insulin deficiency leads to the inability of lipoprotein lipase to perform its physiological functions properly, leading to an increased risk of hyperlipidemia and hypercholesterolemia, as evidenced by increased levels of TG, TC, and LDL-C, along with decreased levels of HDL-C.^29^ In our experiment, the T2DM mice in Cb-GLP-1 group exhibited a significant reduction in serum TG, TC, and LDL-C levels, accompanied by a notable increase in HDL-C levels, indicating superior hypolipidemic effects of Cb-GLP-1 on T2DM. However, it remains unclear whether Cb-GLP-1 exerts anti-dyslipidemia function solely by controlling hyperglycemia or if it directly influences lipid metabolism.

Moreover, the liver is the central site to lipid metabolism. When hepatocytes are damaged or their permeability increases, AST and ALT are released into the bloodstream, leading to elevated serum aminotransferase activity.^30^ The increased serum levels of AST and ALT also are key indicators for assessing the severity of hepatocellular injury.^31^ Here, we observed mice in group M exhibited an increased in serum AST and ALT levels, indicating liver injury in T2DM mice. Interestingly, Cb-GLP-1 demonstrated a liver protective effect by restoring AST and ALT to nearly normal levels, as well as reducing the number of necrotic cells and lipid accumulation in liver (Figure 3).

Studies have reported a marked reduction in pancreatic beta-cell levels and an increased incidence of pancreatic cell apoptosis in T2DM patients compared to the normal control population,^32^ indicating that inhibiting excessive apoptosis of pancreatic islets is crucial for diabetes treatment. PI3K/AKT-mediated signaling pathway has been reported to play a critical role in regulating glucose homeostasis due to its contribution to glucose lipid metabolism and insulin resistance in T2DM subjects.^33–36^ Additionally, the PI3K/Akt signaling pathway is a classic pathway involved in running cell cycle, inhibiting apoptosis, and promoting cell growth and proliferation. To investigate the impact of Cb-GLP-1 on pancreatic apoptosis, we studied morphological changes using H&E staining and analyzed the expression of several proteins in pancreatic tissues, including PI3K, p-PI3K, AKT, p-AKT, Bax, Bcl-2, Caspase-3, and cleaved-Caspase-3, through western blotting. We demonstrated that mice in the group M exhibited evident pancreatic tissues lesions and intensified apoptotic processes, while the Cb-GLP-1 treatment could effectively inhibit pancreatic apoptosis through PI3K/AKT pathway activation and maintain the intact morphological structure of pancreatic islets. The phosphorylation of AKT upon PI3K activation affects the expression of key apoptotic proteins Bax, Bcl-2, and Caspase-3, essential for maintaining cell survival and suppressing apoptosis.^37–40^ The Bax/Bcl-2 ratio determines the occurrence of apoptosis. When Bax is overexpressed in cells, it antagonizes the protective effects of Bcl-2 on cells, which in turn enhances the activation of intracellular caspases, resulting in apoptosis.^41^ Therefore, the downregulated expression ratios of Bax/Bcl-2 and cleaved-Caspase-3/Caspase-3 in the Cb-GLP-1 group in this study suggested that Cb-GLP-1 treatment could inhibit pancreatic cell apoptosis (Figure 4).

Here, we hypothesized that the alleviation of the apoptotic process in pancreatic beta cells might restore their insulin-secreting capacity to some extent. Through western blotting and immunofluorescence detection, we examined the expression of proteins related to insulin- and insulin-secretion-related pathways. The results suggested that the administration of Cb-GLP-1 significantly activated the GLP-1 R/AC/PKA pathway and increased insulin expression. GLP-1, a common intestinal insulinotropic hormone, is able to bind to its receptor GLP-1 R *in vivo* to perform its biological functions.^42^ Subsequently, the activated GLP-1 R stimulates AC to promote the production of cyclic adenosine monophosphate (cAMP), leading to PKA activation.^43^ PDX-1, a transcriptional regulator of the insulin gene in pancreatic beta-cells, is activated in response to this signaling pathway, promoting insulin biosynthesis.^44^ Hence, the upregulation of GLP-1 R, AC, PKA, and PDX-1 protein expression in the Cb-GLP-1 group signifies the critical role of the engineered bacteria in promoting insulin secretion (Figure 5).

Functioning as an indispensable site for nutrient digestion and absorption, the intestinal epithelial barrier plays a crucial role in maintaining intestinal homeostasis, preventing microorganism invasion, and promoting the restoration of intestinal injury, etc.^45^ Research has demonstrated that hyperglycemia can disrupt the integrity of tightly adherent cellular junctions, causing an increase in intestinal barrier permeability.^46^ TJ proteins, the membrane junction complexes between adjacent cells, serve as the essential structural basis for maintaining the mechanical barrier between intestinal mucosal epithelial cells.^47^ In this study, we detected the expression of TJ proteins, particularly Occludin and ZO-1, in colonic tissues by western blotting. We demonstrated that mice in group M exhibited a downregulated expression of Occludin and ZO-1 in their colonic tissues, indicating impaired intestinal barrier function in T2DM mice. Dysfunctional intestinal barriers can result in the infiltration of enteropathogenic microbial metabolites into the bloodstream, contributing to chronic inflammation triggered by metabolic endotoxemia.^48^ Therefore, we also employed qPCR to monitor the transcript levels of colonic pro-inflammatory factors, including TNF-α, IL-6, and IL-1β. As expected, inflammatory levels were elevated in group M. Notably, the Cb-GLP-1 treatment not only exhibited significantly upregulated TJ protein expression but also showed a reduction in transcript levels of colonic inflammatory factors, highlighting its potential in preserving intestinal barrier function (Figure 6).

Accumulating evidence suggests the disturbed gut microbiota is closely associated with colon inflammation and intestinal barrier in T2DM.^49–51^ And the gut microbiota also has been reported to associate with the secretion of enteroendocrine peptides, including GLP-1 produced by L-cells with the putative mechanisms by gut microbiota-derived metabolites, such as short chain fatty acids (SCFAs).^52,53^ And in turn, the application of GLP-1 can improve intestinal barrier functions through stimulating crypt cell fission, downregulating proinflammatory cytokines by immune cells, and modulating gut microbiota.^54^ Therefore, we analyzed the gut microbial alterations in each group by 16S rRNA high-throughput sequencing. The results showed that the intestinal microbiota composition in the Cb-GLP-1 group was closer to that in group C, with a slight reduction in the abundance of the phylum Proteobacteria compared to that in group M. Previous studies have consistently shown a notably higher prevalence of Proteobacteria in T2DM mice than in healthy individuals.^55^ Moreover, the abundance of the genera *Lactobacillus* and *Providencia* was elevated in group M but decreased by Cb-GLP-1 treatment. This aligns with previous research reporting an increase in *Lactobacillus* in the T2DM cohort, where abundance positively correlated with blood glucose levels.^56^ *Providencia*, on the other hand, has been associated with an elevated infection risk in diabetic patients.^57^ Additionally, *Coprococcus*, known for promoting human intestinal health via carbohydrate fermentation, improvement of gastrointestinal functions, and reduction of inflammation,^58^ displayed an increasing trend in the Cb-GLP-1 group (Figure 6). Furthermore, additional association analysis revealed the potential existence of a gut microbiota signature composed of the *Lactobacillus* and *Providencia* genera that may contribute to gut inflammation (Figure S2). This association warrants further clarification in subsequent investigations due to a limitation of a restricted sample size for analysis. Collectively, these results suggest that Cb-GLP-1 may strengthen intestinal epithelial barrier function and thus inhibit inflammation through modulation of the intestinal microecology.

Although the colonization of a probiotic is important for its effectiveness, it is not essential (https://isappscience.org/is-probiotic-colonization-essential/) since probiotics generally do not colonize the digestive tract or other sites on the human body if there is a stable microbiome. As for *C. butyricum* used as probiotics, we did not observe the colonization of *C. butyricum* in the gut both in a clinical trial and rodent animal models, as indicated by non-increased abundance of *Clostridium* genus was identified in the stool samples after administrated with *C. butyricum*.^15,16,59^ Here, we also did not find the increased *Clostridium* genus in the fecal samples using 16S sequencing technique after the administration of *C. butyricum* and its-derived engineering strain, which might further support the non-colonization characteristic of this strain in the gut. In another ongoing work, we demonstrated that the engineered Cb-GLP-1 cannot persistently colonize the GI tract in mice, accompanied with a dynamic fecal GLP-1 and serum GLP-1 levels linked to the content of fecal Cb-GLP-1 (Figure S3). Moreover, from the perspective of biosecurity and bio-contaminant, if it is used to engineer drug delivery system, the genetically engineering probiotic is not advisable to persistently colonize in the intestine and its duration in vivo should be short. Therefore, we proposed that the engineered Cb-GLP-1 showed therapeutic efficacy in improving T2DM in mice as a transient probiotic.

Therefore, we propose a new strategy of delivering GLP-1 through engineered the *C. butyricum* to address the limitation of short half-life of GLP-1. This approach enables the continuous production of GLP-1 in the gut, enhancing GLP-1 levels locally or raising systemic GLP-1 levels. Meanwhile, the chassis itself contributes to gut homeostasis by modulating gut microbiota and metabolism, supporting disease treatment. It also offers significant advantages over other methods, including precise targeting, enhanced safety, controllability, and cost-effectiveness. Specifically, the inherent safety and engineered control of probiotics ensure their potential in disease prevention and treatment. Moreover, eliminating the need for complex purification steps in protein production would substantially reduce future commercialization costs.

In conclusion, our study demonstrated the exceptional hypoglycemic efficacy of the engineered *C. butyricum*-pMTL007-GLP-1 strain in an HFD/STZ-induced T2DM mouse model. This therapeutic effect appears to be achieved by stimulating the expression of proteins involved in the GLP-1 R/AC/PKA insulin pathway. Furthermore, the engineered Cb-GLP-1 possesses a positive treatment role in regulating dyslipidemia, ameliorating liver lesions and islet cell apoptosis, and maintaining the intestinal microbiota balance. These findings provide an innovative perspective and valuable data to support further research and iteration of oral diabetes therapeutics. It is important to note, however, that the effects of butyric acid, a metabolite of *C. butyricum*, have not been investigated in the present study, and the details of how *C. butyricum* synergizes with GLP-1 remain a topic of ongoing research.

## Supplementary Material

Supplemental Material

## Data Availability

All original data are available from the authors on request.
