# Supplementary material for: Hypoglycemic effect of C. butyricum-pMTL007-GLP-1 engineered probiotics on type 2 diabetes mellitus
Source: Gut Microbes. 2025 Jan 2;17(1):2447814. doi: 10.1080/19490976.2024.2447814 (PMC12931707; doi:10.1080/19490976.2024.2447814)
Supplement: Supplemental Material [file KGMI_A_2447814_SM4132.zip › Supplemental material.docx]

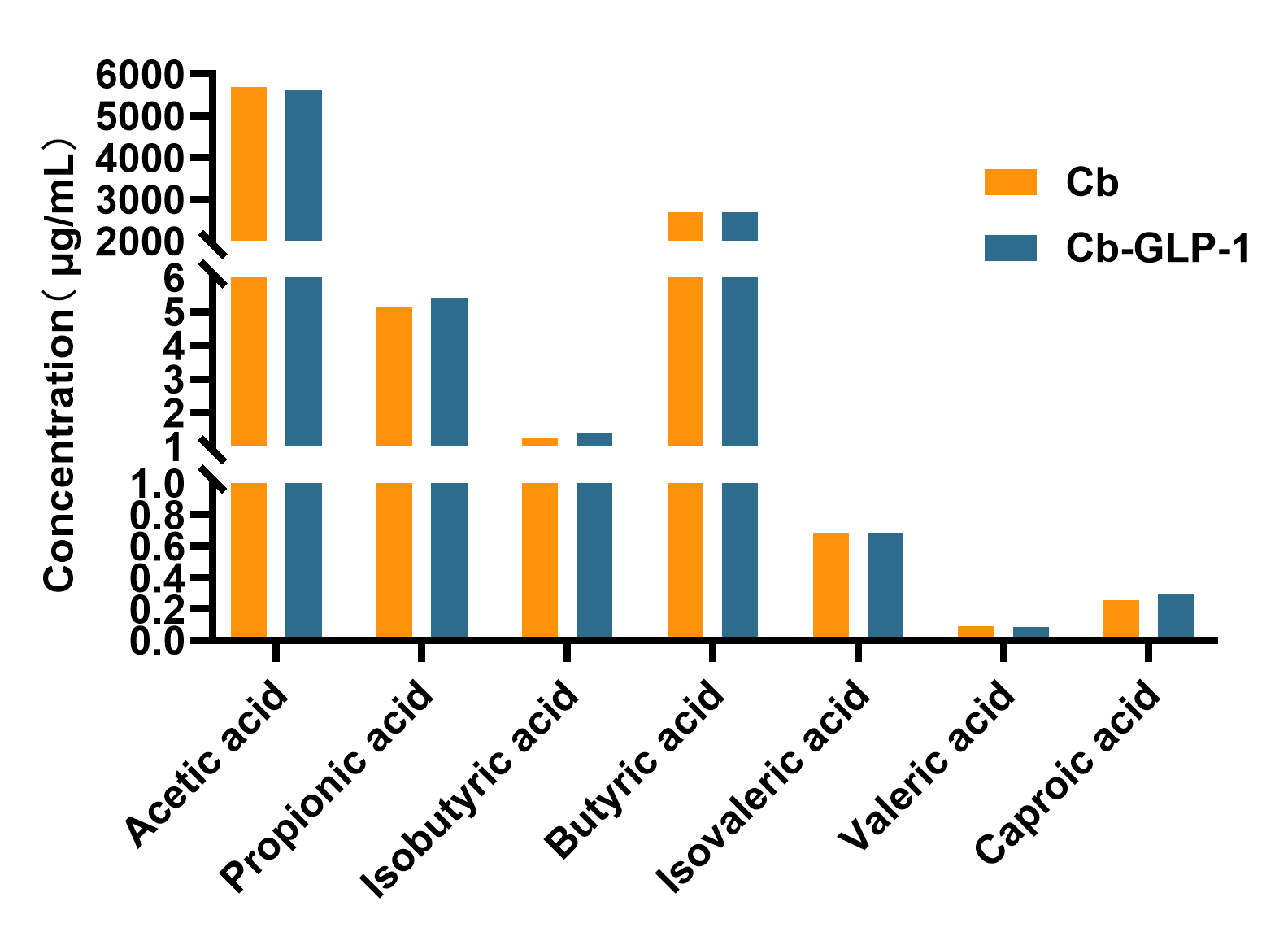


**Figure S1. The ability of Cb-GLP-1 to secrete SCFAs is closely resembled to that of natural bacteria.** Adopt the targeted metabolomics to detect the content of each short-chain fatty acid in bacterial culture supernatants. Cb, *C. butyricum* wild-type strain; Cb-GLP-1, *C. butyricum*-pMTL007-GLP-1 engineered strain.


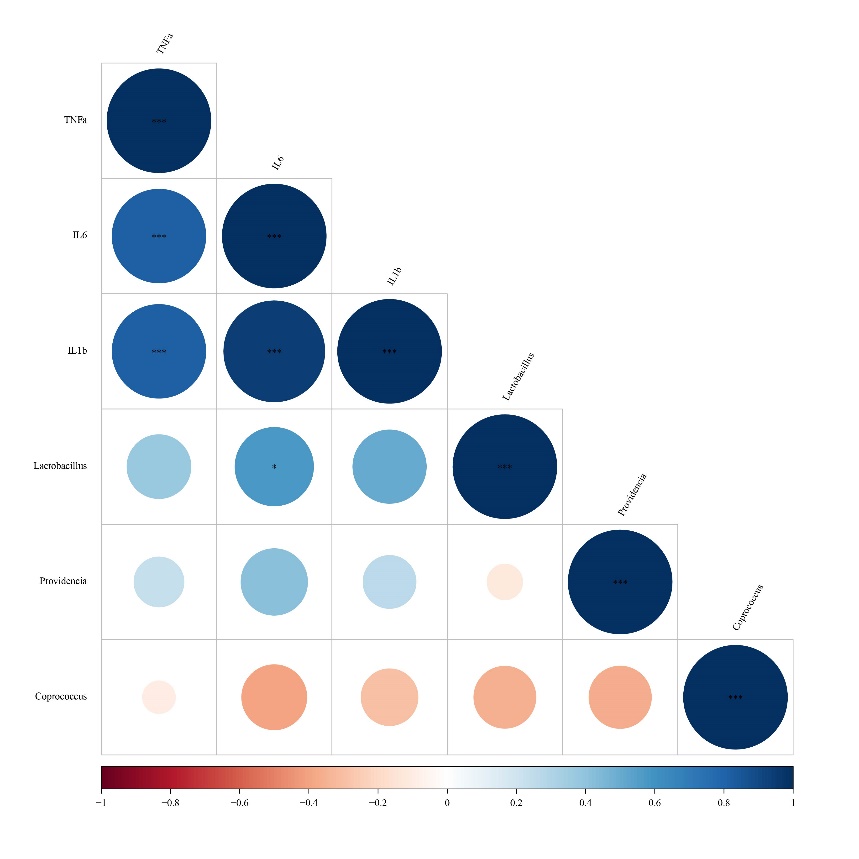


**Figure S2. Spearman correlation analysis between colon proinflammatory factors and the changed gut microbes.** Spearman’s rank correlation coefficient among three inflammatory factors, including TNF-α, IL-6, and IL-1β, and the changed *Lactobacillus*, *Providencia*, and *Coprococcus* genera. *p* values are depicted in red and blue, where red refers to a negative correlation and blue refers to a positive correlation. ∗*p* < 0.05.


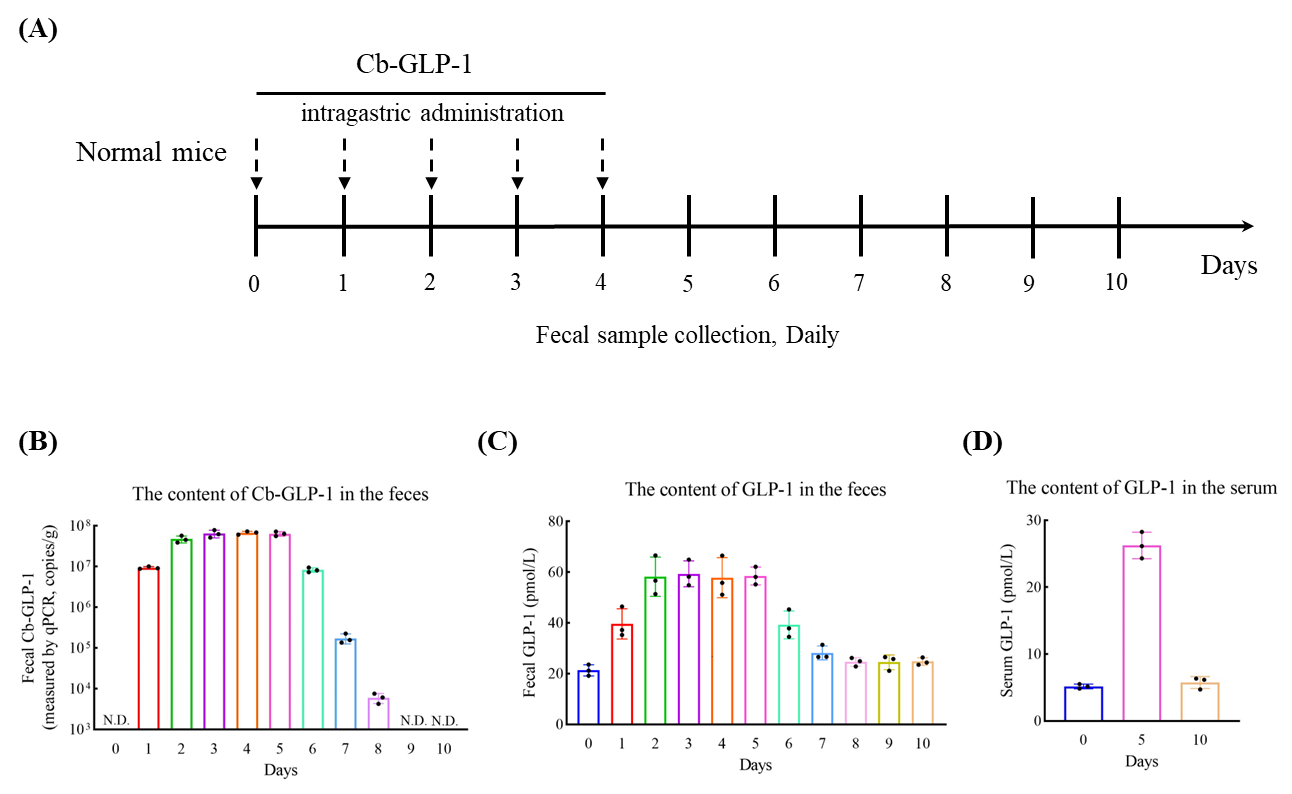


**Figure S3. The colonization of Cb-GLP-1 and its impacts on the levels of fecal GLP-1 and serum GLP-1.** (A) The animal experimental flow chart. Three normal control mice were treated with Cb-GLP-1 by gavage for five times, with fecal samples being collected before each treatment. After final administration, additional fecal samples were collected for six days. Blood samples were also obtained from the tail at day 0, 5, and 10 to measure serum GLP-1 levels. (B) The content of Cb-GLP-1 in the feces was evaluated by qPCR for 10 consecutive days. (C) The content of GLP-1 in the feces was measured by ELISA for 10 consecutive days. (D) The content of serum GLP-1 at day 0, 5, and 10. Cb-GLP-1, *C. butyricum*-pMTL007-GLP-1 engineered strain; N.D., not detect. This supplementary animal experiment is part of an ongoing project approved by the Ethics Committee of Research Involving Animals at Nanchang University (Approval No. NCULAE-20221228055). The project began on December 28, 2022, and is expected to conclude in December 2024.

**Table S1.** Antibodies used for Western blotting

| **Target** | **Provider** | **Catalogue** | **Dilution** |
| --- | --- | --- | --- |
| β-actin | Bioss | bs-0061R | 1:5,000 |
| GLP-1R | HUABIO | ER1909-68 | 1:2,000 |
| AC | Proteintech | 55067-1-AP | 1:1,000 |
| PKA | Proteintech | 27398-1-AP | 1:1,000 |
| PDX-1 | Proteintech | 20989-1-AP | 1:1,000 |
| p-PI3K | CST | 17366s | 1:1,000 |
| PI3K | Abcam | ab151549 | 1:1,000 |
| p-AKT | CST | 4060s | 1:2,000 |
| AKT | CST | 9272s | 1:1,000 |
| Bax | CST | 2772s | 1:1,000 |
| Bcl-2 | CST | 3498s | 1:1,000 |
| Caspase-3 | CST | 9662s | 1:1,000 |
| cleaved-Caspase-3 | Abcam | ab32042 | 1:500 |
| ZO-1 | Abcam | ab96587 | 1:1,000 |
| Occludin | Proteintech | 66378-1-Ig | 1:5,000 |
| Goat Anti-Mouse IgG^HRP^ | CST | 91196s | 1:3,000 |
| Goat Anti-rabbit IgG^HRP^ | CST | 14708s | 1:3,000 |

**Table S2.** Primers for amplifying different genes via q-PCR

| **Target primers** | **Sequence (5’to3’)** |
| --- | --- |
| TNF-α | F: GTGGAACTGGCAGAAGAGGCA |
|  | R: AGAGGGAGGCCATTTGGGAAC |
| IL-6 | F: GAAATCGTGGAAATGAGA |
|  | R: GCTTAGGCATAACGCACT |
| IL-1β | F: GTGTCTTTCCCGTGGACCTTC |
|  | R: TCATCTCGGAGCCTGTAGTGC |
| GAPDH | F: CTCGTGGAGTCTACTGGTGT |
|  | R: GTCATCATACTTGGCAGGTT |
